# PTSD and depressive symptoms in Chinese adolescents exposed to multiple stressors from natural disasters, stressful life events, and maltreatment: A dose-response effect

**DOI:** 10.3389/fpsyg.2022.1050260

**Published:** 2022-12-14

**Authors:** Ting Ni, Yi Zhang, Shuang Xue, Wenjian Xu, Wanjie Tang

**Affiliations:** ^1^College of Environment and Civil Engineering, Chengdu University of Technology, Chengdu, China; ^2^School of Economics and Business Administration, Yibin University, Yibin, China; ^3^Department of Sociology and Psychology, School of Public Administration, Sichuan University, Chengdu, China; ^4^Mental Health Centre, West China Hospital, Sichuan University, Chengdu, China; ^5^King’s College London, Institute of Psychiatry, Psychology and Neuroscience, London, United Kingdom

**Keywords:** PTSD, depression, negative life events, childhood maltreatment, earthquake exposure

## Abstract

**Objectives:**

Little is known about the effects and the extent that childhood adversity has on post-traumatic stress disorder (PTSD) and depression.

**Study design:**

A population-based, epidemiological study from the Wenchuan earthquake.

**Methods:**

A total of 5,195 Wenchuan Earthquake adolescent survivors aged 11–18 years from nine high schools in southwest China completed questionnaires that assessed their PTSD and depression symptoms due to childhood maltreatment, stressful life events, and childhood earthquake exposure.

**Results:**

The PTSD and depression prevalences were 7.1 and 32.4%. After controlling for age and gender, the multiple linear regressions revealed that stressful life events had the most significant direct effect on depression (β = 0.491), followed by childhood emotional abuse (β = 0.085), and earthquake exposure (β = 0.077). Similarly, stressful life events (β = 0.583) were found to have more significant direct effects on PSTD, followed by earthquake exposure (β = 0.140); however, childhood emotional abuse was not found to have an effect. The structural equation modeling (SEM) revealed that there were interactions between the three childhood adversities, with all three concurrently affecting both PTSD and depression.

**Conclusion:**

These findings add weight to the supposition that psychological maltreatment, negative life events, and earthquake exposure contribute to PTSD and depression. In particular, the identification of subgroups that have a high prevalence of these childhood adversities could assist professionals to target populations that are at high risk of mental health problems.

## 1 Introduction

When people experience natural (Wadsworth et al., 2009) or interpersonal trauma (Gardner et al., 2019), they are prone to psychological problems, the most common of which are post-traumatic stress disorder (PTSD) and depression (Ekşi et al., 2007; Espejo et al., 2007; Kessler et al., 2017; McLaughlin et al., 2017; Wang et al., 2023). Teenagers are especially susceptible to mental health problems after trauma because they are not yet physically and mentally developed, which means they tend to have inadequate cognitive and emotional regulation and a poor ability to seek support (Gardner et al., 2019; Guessoum et al., 2020). The dose-response model (Dohrenwend and Dohrenwend, 1974) claims that the greater the trauma, the more severe the mental health problems. Therefore, the mental health of adolescents who have experienced these traumas deserves greater attention.

After experiencing natural disasters, children and adolescents may also experience other human-induced interpersonal traumas, which may together affect the mental health of this vulnerable group (Cénat et al., 2020). For example, in the aftermath of the Haiti earthquake, teenagers who had not fully recovered from the disaster had to deal with interpersonal trauma such as maltreatment (Blanc et al., 2020). While this could also be the case in China, there have been few comprehensive studies; therefore, this area requires further exploration to provide evidence for psychological disaster recovery in children and adolescents.

Exposure to a major earthquake increases the likelihood of both short- and long-term mental health problems, the most frequent of which are PTSD and depression (Norris et al., 2002; Zhang et al., 2010; Kadak et al., 2013; Tang et al., 2017). Although some PTSD symptoms typically appear immediately after the trauma experience, other symptoms may appear 6 months or even later. Some people have been found to remain symptomatic for longer than 12 months, and in some cases, for even more than 50 years (Edition, 2013).

Psychological distress hinders recovery from trauma, especially in children and adolescents. The precise nature of psychological distress after major disasters is not fully understood; however, post-disaster negative life events have been found to be a critical determinant of PTSD and depression in adolescents (Jin et al., 2018; Tang et al., 2020). The greater the number of negative life events experienced in childhood and early adolescence, the greater the vulnerability to depression and PTSD (Phillips et al., 2015; Young and Dietrich, 2015; Pinto et al., 2017; Van Den Berg et al., 2017). Further, children who are maltreated early in life are at a greater risk of poor psychological functions later in their lives (Poole et al., 2017; Frampton et al., 2018; Jones et al., 2018; Van Berkel et al., 2018).

The “Life Change” Model argues that negative life event stressors upset homeostasis and exhaust the coping resources required for readjustment, which makes people more vulnerable to psychopathology (Holmes and Masuda, 1973). Academic pressure has been found to be a major stressor on PTSD symptoms in adolescent survivors of the 2008 Wenchuan earthquake (Zheng et al., 2012), and our previous longitudinal study found that quarreling with parents and peer relationships also predicted PTSD and depression in a sample of adolescent survivors of the Ya’an earthquake (Tang et al., 2017).

There have been four earthquakes of magnitude 6.5 or greater in Sichuan Province, China, over the past two decades, with the latest being the Luding Earthquake with a magnitude of 6.8 on September 4, 2022, in which nearly a hundred people died or were declared missing; therefore, helping young people in earthquake-stricken areas with their psychological rehabilitation is particularly important. Children and adolescent earthquake survivors face not only the impacts of earthquake exposure but are also at risk of maltreatment within the family, especially emotional abuse and neglect. Similar to physical abuse, child maltreatment, which is often culturally tolerated in China (Chen and Chan, 2016), can lead to poor mental health. Only a few studies have examined the extent to which child maltreatment after a natural traumatic event contributes to PTSD or depression. For example, sexual assault, but not the earthquake exposure itself, was found to have a significant association with PTSD in secondary school students in Haiti 3 years after the disaster (Grelotti et al., 2018). A cross-sectional study into four maltreatment types; physical abuse, physical neglect, emotional abuse, and emotional neglect; found that emotional abuse and neglect had significant positive correlations with delayed-expression PTSD and depression in adolescent survivors 3 years after the Ya’an earthquake (Tang W. et al., 2018). Early childhood adversity has also been found to be highly correlated with later negative life events, and exposure to childhood adversity has been associated with significantly elevated socially prescribed perfectionism and perfectionistic self-presentation styles (Chen et al., 2019). Adolescents who experienced childhood abuse have been found to be more vulnerable to negative life events. For example, a prospective study of 705 adolescents who experienced childhood trauma in Australia found that trauma experienced at age five correlated with negative life events at age 15 and affected depression levels at and after age 15 because of the total number of stress events that had been experienced (Hazel et al., 2008).

To further explore the relationship between negative factors, PTSD, and depression, this study assessed the influences of negative life events, child maltreatment, and earthquake experiences on PTSD and depression prevalence in a large group of adolescent survivors 8.5 years after the 2008 Wenchuan earthquake (Richter magnitude 8.0). This study is particularly meaningful because earthquake survivors could have experienced concurrent traumas, such as negative life events and family adversity (Ip et al., 2016; Liu and Wang, 2018; Cui and Liu, 2020), which could have confounded the factor contributions to their post-disaster psychological distress. Therefore, the following three hypotheses were tested: (Wadsworth et al., 2009). As a function of age and gender, PTSD and depression differ in adolescents who have experienced multiple difficulties (Gardner et al., 2019); earthquake exposure, negative life events, and child maltreatment are related to the depressive and/or PTSD symptoms in adolescents; and (Wang et al., 2023) earthquake exposure, negative life events, and child maltreatment are correlated.

## 2 Materials and methods

### 2.1 Participants and procedure

Between October and November 2016, participants were recruited for a cross-sectional study using convenience sampling in Hanyuan and Shimian counties in Sichuan province, both of which had been severely affected by the 2008 Wenchuan Earthquake (Liu et al., 2016; Chang et al., 2020). Ten suitable junior and senior high schools in the two counties were contacted through the local Education Bureau, of which one senior high school and eight junior high schools agreed to participate. Cluster sampling was conducted by class, which involved 5,413 adolescents. To be enrolled in the study, the participants had to be attending one of the participating schools and had to be aged 11–18 years at the time of the study. However, 186 participants were excluded from the analyses because they failed to complete all questionnaires or their responses showed systematic bias (e.g., all responses were “yes” or “no”). A further 32 were excluded because they were adults (age > 18 years); finally, 5,195 adolescents were included in the data analyses.

This study was approved by the Research Ethics Committee of our Institute, the Departments of Education of Hanyuan and Shimian Counties, and the principals and management teams at the participating schools. Parents and children were provided with written informed consent before enrollment in the study, students were fully informed of the study’s purpose, and the voluntary and confidential nature of their participation was emphasized. The data were collected through surveys that were administered in class. The paper and pencil questionnaires were distributed to the students through the teacher in charge, and our research assistants (trained Master’s level students majoring in psychology) were responsible for explaining the survey questions.

### 2.2 Post-traumatic psychological measurement scales

The Children’s Revised Impact of Event Scale (CRIES) has 13 items that assess child and adolescent PTSD symptoms, such as intrusion, avoidance, and hyper-arousal (Perrin et al., 2005). In this study, the Chinese version of the scale was used to measure the stress symptoms of the adolescents in response to the 2008 Wenchuan earthquake. A sample item was, “Do you stay away from reminders of the 2008 Wenchuan earthquake?” Answers were measured on a 4-point Likert scale (0 = not at all, 1 = rarely, 3 = sometimes, 5 = often), with the possible total score ranging from 0 to 65. Higher scores indicated a greater degree of PTSD symptoms, and a total score of 30 or higher was deemed indicative of probable PTSD (Perrin et al., 2005). The scale for the Chinese version has proven reliable in Chinese adolescent samples (Jin et al., 2012; Lau et al., 2013; Hu et al., 2021). Cronbach’s α for the scale was 0.879.

The abbreviated Chinese version of the Kutcher Adolescent Depression Scale is a 6-item self-report questionnaire (LeBlanc et al., 2002) with all responses given on a 4-point Likert scale (0 = hardly ever, 1 = much of the time, 2 = most of the time, and 3 = all of the time). The total score ranges from 0 to 18, with higher scores indicating possible depressive symptoms and a cutoff score of 6 taken as identifying probable depression (LeBlanc et al., 2002). This scale has demonstrated good psychometric properties in Chinese children and adolescents (Chung et al., 2011). In this study, Cronbach’s alpha was 0.864.

The Adolescent Self-Rating Life Events Checklist (ASLEC) is a 27-item self-report questionnaire that documents exposure to a broad range of stressful life events (Liu et al., 1997). ASLEC comprises five subscales; interpersonal difficulties, academic pressure, being punished, personal loss, health and adaptability; in which there are 26 questions about stressful life events in the preceding 12 months related to school, personal relationships, physical diseases, family, and other domains of life, which respondents answer on a 5-point rating system (1 = not at all to 5 = severely bothered). A 27th item also asks respondents to describe any additional stressful events. The Chinese version of the ASLEC has been found to have satisfactory test-retest reliability and internal consistency (Geng et al., 2013). In this study, Cronbach’s alpha for the whole scale was 0.909, and for the five subscales were 0.835 (interpersonal difficulties), 0.715 (academic pressure), 0.799 (being punished), 0.768 (personal loss), and 0.743 (health and adaptability).

The Childhood Trauma Questionnaire (CTQ) is a 28-item self-report measure that assesses the occurrence and extent of abuse and neglect. Participants rate the frequency of their experiences during their lives on a 5-point scale from 0 (never true) to 5 (very often true). This Chinese version has also been proven reliable and valid in Chinese adolescent populations (Li et al., 2014). The original CTQ comprised five subscales: sexual abuse, physical abuse, physical neglect, emotional neglect, and emotional abuse; however, the sexual abuse subscale was deleted at the request of school personnel and parents. In this study, Cronbach’s alpha for the total scale was 0.872, and for the four subscales was 0.724 (emotional abuse), 0.794 (emotional neglect), 0.812 (physical abuse), and 0.722 (physical neglect).

The severity of earthquake exposure experiences was measured using a self-report questionnaire based on an 8-item scale (Roussos et al., 2005; Tang F. et al., 2018) that included objective and subjective traumatic experience features: feeling extremely scared; being trapped; being injured; having parents, relatives or friends who were injured; witnessing people trapped; witnessing bloody injuries; suffering the loss of a loved one; and witnessing death. All questions were coded as yes/no answers.

### 2.3 Statistical analyses

SPSS 22.0 for Windows (IBM, Chicago, IL, USA) was used to analyze the data, for which the significance level was set at 0.05. Descriptive statistics were computed for the categorical variables along with the proportions exceeding the clinical cutoff. A chi-squared test was conducted to examine the frequency differences for probable PTSD and depression in the different age and gender groups. Hierarchical multiple regressions in which age and gender were controlled were also used to assess whether the different traumatic events; earthquake exposure, negative life events, and child maltreatment; were significantly related to PTSD or depression, and the variance at each step was examined. Structural equation modeling (SEM) was constructed using the AMOS 20.0 program to examine the relationships between the factors influencing depression and PTSD that had been screened by the regression analysis. The comparative fit index (CFI), the Tucker-Lewis index (TLI), the normed fit index (NFI), and the incremental fit index (IFI) ≥ 0.90, and the root mean square error of approximation (RMSEA) < 0.08 were employed to assess the optimal fit of the data to the hypothesized model.

## 3 Results

### 3.1 Sociodemographic and overall psychological examination results

Of the 5,195 participants, 2,652 were male (51.0%), and the mean age was 14.03 ± 1.39 years. The PTSD prevalence was 7.1%, and the depression symptom prevalence was 32.4%. The average total ASLEC score was 32.6 (*SD* = 19.24) and the average CTQ-23 score was 31.05 (*SD* = 9.77). The mean values for the measured parameters are shown in Table 1.

**TABLE 1:** Descriptive statistics for post-traumatic psychological evaluations from Chinese adolescents experiencing multiple difficulties (*N* = 5,195).

Measure	Mean ± SD or *n* (%)	Range	Clinical cut-off	% over clinical cutoff
Age	14.03 ± 1.39	11–18	N/A	N/A
Female	2,543 (49.0)	N/A	N/A	N/A
Depression symptoms	4.51 ± 3.11	0–18	6	1,681 (32.4)
PTSD	10.80 ± 11.10	0–65	30	369 (7.1)
Negative life events	32.6 ± 19.24	0–135	N/A	N/A
Interpersonal difficulties	7.95 ± 4.82	N/A		
Academic pressure	10.66 ± 5.95	N/A		
Being punished	6.26 ± 5.85	N/A		
Personal loss	3.44 ± 3.17	N/A		
Health and adaptability	3.71 ± 3.14	N/A		
Maltreatment	31.05 ± 9.77	0–100	N/A	N/A
Emotional abuse	7.14 ± 2.87	N/A		
Emotional neglect	10.08 ± 5.16	N/A		
Physical abuse	6.10 ± 2.44	N/A		
Physical neglect	7.72 ± 2.88	N/A		
Earthquake exposures	1.60 ± 1.58	0–8	N/A	N/A

N/A, not applicable; PTSD, post-traumatic stress disorder.

### 3.2 Probable PTSD and depression stratified by age and sex

The probable PTSD or depression prevalences did not differ significantly between the male and female adolescents (Table 2). However, the PTSD prevalence was significantly lower in students aged 11–14 years (6.0%) than in students ages 15–16 and 17–18 (9.1 and 10.2%, respectively, χ^2^ = 19.67). Similarly, the depression symptom prevalence was significantly lower in students aged 11–12 years (28.7%) and 13–14 years (29.4%) than in students aged 15–16 years and 17–18 years (36.5 and 48.7%, respectively, χ^2^ = 60.94).

**TABLE 2:** PTSD and depression in adolescents experiencing multiple difficulties (*n* = 5,195) stratified by age and gender.

Variable		PTSD			Depression		
	*N*	*n*	%	χ^2^	*n*	%	χ^2^
Total	5,195	369	7.1		1,681	32.4	
Age group				19.67***			60.94***
11–12 yr	665	40	6.0		191	28.7	
13–14 yr	2,763	165	6.0		811	29.4	
15–16 yr	1,492	136	9.1		545	36.5	
17–18 yr	275	28	10.2		134	48.7	
Gender				1.79			0.01
Male	2,652	176	6.6		857	32.3	
Female	2,543	193	7.6		824	32.4	

PTSD, post-traumatic stress disorder.

***p < 0.001.

### 3.3 Factors correlated with depression and PTSD

Multiple linear regressions were conducted to test whether the earthquake trauma, negative life events, or child maltreatment variables were significantly related to depression when age and gender were controlled (Table 3). The following variables were found to be related to depression: negative life events (Δ*R*^2^ = 0.13) including academic pressure (β = 0.24), interpersonal difficulties (β = 0.17), health and adaptability (β = 0.10); earthquake exposure (β = 0.10) (Δ*R*^2^ = 0.03); and maltreatment (Δ*R*^2^ = 0.10), which was mainly related to emotional abuse (β = 0.14).

**TABLE 3:** Hierarchical multiple regression to identify the factors associated with depressive symptoms in adolescents experiencing multiple difficulties (*N* = 5,195)^a^.

Predictor	Δ*R*^2^	B	*SE*	β	*t*	*p*
Step 1:	0.01					< 0.001
Age		0.47	0.06	0.11	8.25	< 0.001
Gender		0.11	0.09	0.02	1.26	0.21
Step 2:	0.03					< 0.001
Age		0.41	0.06	0.10	7.19	< 0.001
Gender		0.14	0.09	0.02	1.67	0.09
Earthquake exposures		0.34	0.03	0.17	12.47	< 0.001
Step 3:	0.10					
Age		0.31	0.05	0.07	5.67	< 0.001
Gender		0.15	0.08	0.02	1.80	0.07
Earthquake exposures		0.27	0.03	0.14	10.35	< 0.001
Emotional abuse		0.32	0.02	0.30	19.17	< 0.001
Emotional neglect		0.02	0.01	0.03	1.72	0.09
Physical abuse		–0.03	0.02	–0.02	–1.60	0.11
Physical neglect		0.06	0.02	0.06	3.60	< 0.001
Step 4:	0.13					
Age		0.03	0.05	0.01	0.56	0.58
Gender		0.06	0.08	0.01	0.78	0.43
Earthquake exposures		0.20	0.02	0.10	8.15	< 0.001
Emotional abuse		0.15	0.02	0.14	9.17	< 0.001
Emotional neglect		0.03	0.01	0.05	3.35	0.001
Physical abuse		–0.04	0.02	–0.03	–2.37	0.02
Physical neglect		0.04	0.02	0.03	2.22	0.03
Interpersonal difficulties		0.11	0.01	0.17	9.36	< 0.001
Academic pressure		0.13	0.01	0.24	14.05	< 0.001
Being punished		–0.01	0.01	–0.02	–0.89	0.37
Personal loss		–0.06	0.02	–0.06	–4.00	< 0.001
Health and adaptability		0.10	0.02	0.10	5.74	< 0.001

^a^Dependent variable, depressive symptoms; B, unstandardized beta weight; SE, standard error.

A similar step-wise regression analysis was performed to identify the variables related to PTSD (Table 4). The same variables were found to be related to PTSD as for depression: negative life events (Δ*R*^2^ = 0.18) including academic pressure (β = 0.22), interpersonal difficulties (β = 0.13), health and adaptability (β = 0.14); earthquake exposure (β = 0.14) (Δ*R*^2^ = 0.05); and maltreatment (Δ*R*^2^ = 0.09), which was mainly associated with emotional abuse (β = 0.10).

**TABLE 4:** Hierarchical multiple regression analysis to identify the factors associated with PTSD in adolescents experiencing multiple difficulties (*N* = 5,195)^a^.

Predictor	Δ*R*^2^	B	*SE*	β	*t*	*p*
Step 1:	0.02					< 0.001
Age		1.88	0.21	0.13	9.18	< 0.001
Gender		0.95	0.31	0.04	3.11	0.002
**Step 2:**	0.05					< 0.001
Age		1.56	0.20	0.11	7.81	< 0.001
Gender		1.11	0.30	0.05	3.74	< 0.001
Earthquake exposures		1.63	0.10	0.23	17.24	< 0.001
Step 3:	0.09					
Age		1.26	0.19	0.08	6.55	< 0.001
Gender		1.06	0.29	0.05	3.67	< 0.001
Earthquake exposures		1.37	0.09	0.19	15.00	< 0.001
Emotional abuse		1.06	0.06	0.27	17.87	< 0.001
Emotional neglect		–0.13	0.04	–0.06	–3.87	< 0.001
Physical abuse		0.17	0.07	0.04	2.48	0.01
Physical neglect		0.14	0.06	0.04	2.26	0.02
Step 4:	0.18					
Age		0.07	0.17	0.01	0.41	0.68
Gender		0.81	0.26	0.04	3.12	0.002
Earthquake exposures		0.99	0.08	0.14	12.20	< 0.001
Emotional abuse		0.40	0.06	0.10	7.10	< 0.001
Emotional neglect		–0.08	0.03	–0.04	–2.51	0.01
Physical abuse		0.05	0.06	0.01	0.88	0.38
Physical neglect		0.03	0.06	0.01	0.51	0.61
Interpersonal difficulties		0.29	0.04	0.13	7.04	< 0.001
Academic pressure		0.41	0.03	0.22	13.35	< 0.001
Being punished		0.01	0.03	0.01	0.30	0.77
Personal loss		0.30	0.05	0.09	5.89	< 0.001
Health and adaptability		0.49	0.06	0.14	8.47	< 0.001

^a^Dependent variable, PTSD; PTSD, post-traumatic stress disorder; B, unstandardized beta weight; SE, standard error.

### 3.4 SEM

**FIGURE 1:**
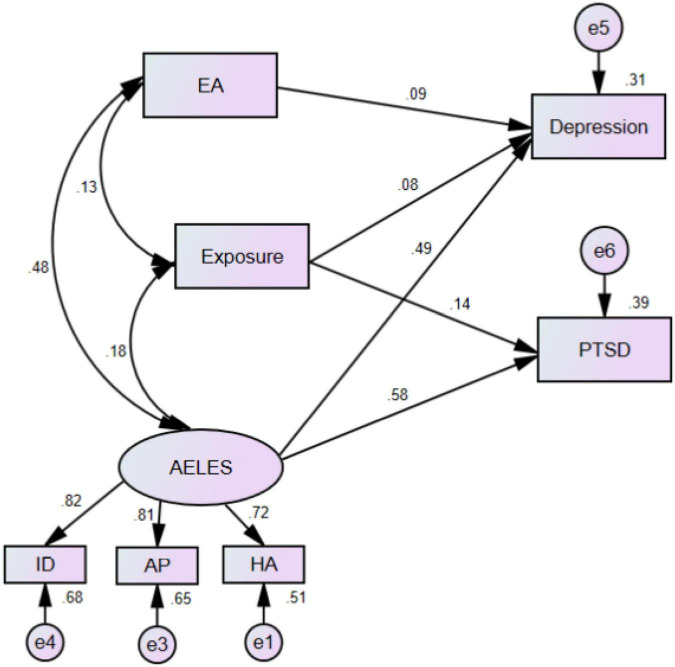
Structural equation modeling model and standardized model path. PTSD, post-traumatic stress disorder; EA, emotional abuse; AELES, adolescent life events; ID, interpersonal difficulties; AP, academic pressure; HA, health and adaptability. “Exposure” refers to earthquake-related exposure.

A SEM analysis was then conducted using the adversity factors influencing PTSD and depression symptoms identified in the multiple linear regressions. The overall model fit indices for the modified hypothetical model were NFI = 0.983, TLI = 0.954, CFI = 0.983, IF = 0.984, and RMSEA = 0.075, all of which satisfied the reference values and suggested that it was an acceptable model fit. The significant standardized path coefficients for the model are shown in Figure 1. The SEM models revealed that childhood emotional abuse, earthquake exposure, and stressful life events (including interpersonal difficulties, academic pressure, health and adaptability) were all related to depression. Stressful life events were found to have the most significant direct effect on depression (β = 0.491), followed by childhood emotional abuse (β = 0.085), and earthquake exposure (β = 0.077). Of these adversities, stressful life events (β = 0.583) and earthquake exposure (β = 0.140) were also observed to have significant direct effects on PSTD; however, childhood emotional abuse was not found to have any effect. Correlations between these three adversities were also observed: stressful life events correlated with childhood emotional abuse (β = 0.482), stressful life events with earthquake exposure (β = 0.185), and childhood emotional abuse with earthquake exposure (β = 0.128).

## 4 Discussion

Epidemiological studies have consistently found that exposure to multiple traumatic events is quite common (Kessler et al., 1995; Kilpatrick et al., 2013), and many studies have also explored the predictive role played by poly-victimization or multiple traumatic events on adolescent psychopathology (Suliman et al., 2009; Ying et al., 2013; Adams et al., 2016). However, this study extended these previous studies by comprehensively and systematically exploring the mental health consequences in a large representative adolescent sample 10 years after the Wenchuan earthquake, which expanded the understanding of the relationships between multiple traumatic events; previous earthquake exposure, negative life events, and child maltreatment; and PTSD and depression in adolescents. The major findings were as follows. First, the probable PTSD prevalence was 7.1% and the probable depression prevalence was 32.4% in the sample of 5,195 adolescents who had experienced multiple difficulties. Second, age, but not gender, was found to significantly affect the risk of PTSD and depression symptoms; however, this effect disappeared when more covariables were added to the regression model. Third, negative life events; academic pressure, health and adaptability, and interpersonal difficulties; were the largest influencing factors for adolescent PTSD and depression. Fourth, correlations were found between stressful life events, earthquake exposure, and childhood emotional abuse.

This study revealed that after controlling for demographics, the three negative life event subcategories; academic pressure, health and adaptability, and interpersonal difficulties; significantly contributed to the variances in PTSD and depressive symptoms. Academic pressure was found to be a powerful predictor of PTSD, which was consistent with previous studies on adolescents who had only experienced earthquake trauma (Zheng et al., 2012). The present study extends the literature by providing evidence that in addition to natural disaster experiences, academic pressure can also contribute significantly to PTSD and depression. In China, academic pressure has often been associated with mental health problems, including sleeping difficulties (Liu et al., 2000), anxiety and depression (Quach et al., 2015), and suicidal risk (Liu and Tein, 2005; Tang W. et al., 2018). Academic achievement has been historically emphasized in China because of the cultural values that link education with personal success and social status (Hesketh et al., 2005; Shek et al., 2013). The combination of strict school regulations, the one-sided pursuit of academic achievement, fierce peer competition, and high parent expectations make adolescent students feel frustrated, helpless, and exhausted, and therefore vulnerable to mental health problems (Tang et al., 2015). Therefore, it is not surprising that Chinese students feel pressure to excel academically at the expense of their psychological and emotional health.

Health, adaptability, interpersonal difficulties, and earthquake exposure severity all contribute to PTSD and depression prevalence. This study provides evidence that earthquake trauma still impacts adolescent mental health even many years later. These findings are also in line with other trauma studies that found a direct connection between trauma severity and the victims’ psychological distress (Horesh et al., 2011; Du et al., 2018). Negative events associated with health and adaptability may serve as reminders and trigger emotional distress, especially PTSD symptoms. A connection between interpersonal difficulties and depression fits within Coyne’s interpersonal theory of depression, that is, relationship difficulties can give rise to depression, which in turn leads to a vicious cycle of aversive interpersonal behaviors that exacerbate interpersonal difficulties (Coyne, 1976). Therefore, adolescent interpersonal difficulties may exacerbate the emotional disturbances resulting from earthquake exposure and increase the risk of depression. Taken together, these results suggest that programs that assist adolescent survivors of natural disasters to solve interpersonal problems and reduce their worries about their health and adaptability to school life could help mitigate psychological distress.

These results provide strong evidence that emotional abuse, one maltreatment subtype, may also exacerbate depression, which was in line with our previous study that found that emotional abuse increased adolescent suicide risk through direct and indirect paths mediated by depression (Tang W. et al., 2018). The present study also found that emotional abuse, such as threatening, demeaning, terrorizing, or humiliating remarks or behavior directed at a child by an older person that challenges a child’s sense of wellbeing or self-worth (Glaser, 2002), had a significant effect on depression (Schulz et al., 2017; Berzenski et al., 2019). At least one in five Chinese adolescents experience emotional abuse (Fang et al., 2015; Di et al., 2018; Wan et al., 2019). These results may partly reflect traditional Chinese parenting styles, which are often described as “authoritarian,” “controlling,” or “restrictive.”

The SEM found that stressful life events; academic pressure, health and adaptability, and interpersonal difficulties; correlated with childhood emotional abuse (β = 0.482), which indicated that individuals with childhood trauma were more vulnerable to negative life events. The reason for this might be that early trauma is often accompanied by disharmony between parents, poverty, and other family problems, which together can result in highly stressful family environments. The persistence of such an environment could increase the risk of recent negative life events (Hammen, 2005). As people who have suffered from early trauma are more sensitive to later negative life events, new negative events may trigger a recall of earlier trauma, which puts further pressure on them and increase the adverse effects of these negative life events on their psychology (Comijs et al., 2013; Shapero et al., 2014). The finding that older adolescents were more likely to have PTSD and depressive symptoms could also indicate a positive relationship between academic pressure and age in the Chinese culture (Zhao et al., 2015). These findings highlight the need for school- and family-based care as well as preventive and therapeutic strategies in early childhood to prevent the development of mental health problems, especially in areas susceptible to natural disasters where there is a higher risk of traumatic experiences.

This study had some limitations. First, it was based on self-report measures that although very common in trauma studies, have the risk of recall and reporting bias. Second, the cross-sectional design was unable to capture the time-dependent fluctuations in PTSD and depression and allow for any conclusions to be drawn on the cause-effects. Third, other potential risk factors, such as pre-trauma stressors, personality type, and social support, were not examined. Finally, a more detailed measurement of stressful life events (e.g., time of occurrence) could have provided a clearer explanation for the possible influences of stressful events on adolescent PTSD and Depression symptoms.

## 5 Conclusion

In summary, a dose-effect association was found between the number of trauma events and the psychological consequences. This study found that academic pressure, interpersonal difficulties, health and adaptability, emotional abuse, and earthquake exposure all increased the risk of depression in adolescents. Except for emotional abuse, these factors were found to increase the risk of PTSD; however, emotional abuse and stressful life events were found to be strongly correlated. These findings strengthen the belief that psychological maltreatment, negative life events, and earthquake exposure all contribute to PTSD and depressive symptoms (Cerdá et al., 2013; Derivois et al., 2017; Grelotti et al., 2018). In particular, the identification of the subgroups that have a high prevalence to these stressors could assist professionals to target the populations at a higher risk of mental health problems. However, it should be noted that there is still some debate on this point; for instance, recent research has challenged the assumption that screening for adverse life events can give accurate information on mental health risk predictions due to retrospective recall bias, pre-existing vulnerabilities, or conflicting results between studies (Baldwin and Danese, 2021). Therefore, when seeking to apply the results of this study, the influence of stress sensitization (McLaughlin et al., 2010; Smid et al., 2012; Stroud et al., 2020) and vulnerability traits (Elwood et al., 2009) should also be considered. It is also necessary to consider post-traumatic growth (Chen et al., 2022; Jian et al., 2022) and the positive effects of experiencing trauma (Lahav et al., 2020), which may counterbalance any psychopathology. Finally, this study highlighted the potential importance of age-specific psychological interventions to relieve academic pressure; however, the effect only exists if the full model is not considered. Therefore, more research is needed to clarify this age effect on post-trauma mental health problems, and longitudinal research is also needed to clarify the effects of adolescent experiences on their susceptibility to depression and PTSD after multiple traumas.

## Data availability statement

The original contributions presented in this study are included in the article/supplementary material, further inquiries can be directed to the corresponding author.

## Ethics statement

The studies involving human participants were reviewed and approved by the Research Ethics Committee of Sichuan University. Written informed consent to participate in this study was provided by the participants’ legal guardian/next of kin.

## Author contributions

TN, YZ, SX, WX, and WT wrote the main manuscript text. SX prepared the tables. TN prepared the figure. All authors contributed to the article and approved the submitted version.
